# SLAP Is a Negative Regulator of FcεRI Receptor-Mediated Signaling and Allergic Response

**DOI:** 10.3389/fimmu.2019.01020

**Published:** 2019-05-15

**Authors:** Namit Sharma, Marta Ponce, Savar Kaul, Zhongda Pan, Donna M. Berry, Thomas Eiwegger, Catherine J. McGlade

**Affiliations:** ^1^Program in Cell Biology and the Arthur and Sonia Labatt Brain Tumour Research Centre, The Hospital for Sick Children, Toronto, ON, Canada; ^2^Program in Translational Medicine, The Hospital for Sick Children, Toronto, ON, Canada; ^3^Department of Pediatrics and Adolescent Medicine, Medical University of Vienna, Vienna, Austria; ^4^Department of Immunology, University of Toronto, Toronto, ON, Canada; ^5^Department of Medical Biophysics, University of Toronto, Toronto, ON, Canada; ^6^Food allergy and Anaphylaxis Program, Division of Immunology and Allergy, Department of Paediatrics, The Hospital for Sick Children, Toronto, ON, Canada

**Keywords:** IgE receptor, mast cell, basophil, signaling, Cbl-b, SLAP

## Abstract

Binding of antigen to IgE-high affinity FcεRI complexes on mast cells and basophils results in the release of preformed mediators such as histamine and *de novo* synthesis of cytokines causing allergic reactions. Src-like adapter protein (SLAP) functions co-operatively with c-Cbl to negatively regulate signaling downstream of the T cell receptor, B cell receptor, and receptor tyrosine kinases (RTK). Here, we investigated the role of SLAP in FcεRI-mediated mast cell signaling, using bone marrow derived mast cells (BMMCs) from SLAP knock out (SLAP KO) mice. Mature SLAP-KO BMMCs displayed significantly enhanced antigen induced degranulation and synthesis of IL-6, TNFα, and MCP-1 compared to wild type (WT) BMMCs. In addition, SLAP KO mice displayed an enhanced passive cutaneous anaphylaxis response. In agreement with a negative regulatory role, SLAP KO BMMCs showed enhanced FcεRI-mediated signaling to downstream effector kinases, Syk, Erk, and Akt. Recombinant GST-SLAP protein binds to the FcεRIβ chain and to the Cbl-b in mast cell lysates, suggesting a role in FcεRI down regulation. In addition, the ubiquitination of FcεRIγ chain and antigen mediated down regulation of FcεRI is impaired in SLAP KO BMMCs compared to the wild type. In line with these findings, stimulation of peripheral blood human basophils with FcεRIα antibody, or a clinically relevant allergen, resulted in increased SLAP expression. Together, these results indicate that SLAP is a dynamic regulator of IgE-FcεRI signaling, limiting allergic responses.

## Introduction

Antigen induced crosslinking of IgE, bound to high affinity FcεRI on the surface of mast cells (MCs) and basophils, results in the degranulation and release of mediators including histamine as well as *de novo* synthesis of pro-inflammatory cytokines, growth factors and chemokines (1, 2). FcεRI-mediated signaling involves cytoplasmic tyrosine kinases and adapter molecules, that can both positively and negatively regulate the response to antigen. Upon antigen exposure, signaling initiates with tyrosine phosphorylation of ITAM motifs in FcεRI by the Src-family kinase Lyn and subsequent recruitment of Syk tyrosine kinase. The transmembrane protein tyrosine phosphatase CD45 plays a role in the initiation of FcεRI signaling, by dephosphorylating the carboxy-terminal negative regulatory tyrosine site in Lyn. Activated Lyn and Syk phosphorylates the downstream effector molecule LAT resulting in the recruitment of positive signaling mediators, such as PLCγ, Gads/SLP-76/Vav, and Grb/Shc/Sos, as well as intracellular calcium mobilization and degranulation (3, 4). Activated FcεRI receptors also initiate Gab2-Fyn-PI3Kinase signaling and MAP kinase-NFκB signaling that regulates calcium mobilization, degranulation and production of cytokines including TNFα, IL-6 and MCP-1 (4, 5). To balance the FcεRI signaling response, several negative regulators are engaged including the SH2 domain-containing protein tyrosine phosphatase 1 (SHP1), the lipid phosphatases, SH2 domain-containing inositol polyphosphate 5-phosphatase (SHIP) and Phosphatase and tensin homolog (PTEN), as well as inhibitory receptors such as FcγRIIb and the mast function-associated antigen (MAFA) (3). Additional regulatory mechanisms involve ubiquitin ligases Cbl and Cbl-b that promote proteasomal degradation of substrates and receptor internalization, thereby dampening the signal (6).

The Cbl family of ubiquitin ligases is involved in the regulation of tyrosine kinase mediated signaling including the activated T cell receptor (TCR), B cell receptor (BCR), and growth factor receptors (7, 8). The Cbl family is comprised of three genes encoding c-Cbl, Cbl-b and Cbl-c. Previous studies have shown that Cbl-b is the major isoform expressed in murine MCs and Cbl-b null MCs have reduced FcεRI internalization, enhanced downstream signaling and enhanced mast cell functions including degranulation and production of TNFα, IL-6, and MCP-1 (9, 10).

In the context of BCR and TCR signaling, Cbl function requires the SH2 domain and SH3 domain containing Src-like adapter protein (SLAP). SLAP regulates TCR signaling via the recruitment of c-Cbl leading to ubiquitination and degradation of TCR ζ chains (11, 12). Similarly, SLAP alters BCR recycling and promotes receptor down regulation via the recruitment of c-Cbl (13). SLAP has also been shown to regulate the cell surface levels of the c-Kit and Flt3 receptor tyrosine kinases via ubiquitin mediated degradation, and SLAP null dendritic cells have enhanced the GM-CSF signaling and elevated GM-CSF receptor expression (14–16). Together these studies support the role of SLAP as a negative regulator of tyrosine kinase mediated signaling in hematopoietic cells. A previous study demonstrated that dexamethasone, often used in the treatment of MC mediated allergic responses, causes the up regulation of SLAP mRNA, suggesting that SLAP may also play a role in MC mediated allergic responses (17). Here we report that SLAP functions as a negative regulator of degranulation and cytokine secretion *in vitro* and *in vivo*. SLAP deficient MCs show enhanced signaling and we provide evidence that Cbl-b function is altered in the absence of SLAP. Furthermore, FcεRI activation in human basophils from allergic patients results in elevated levels of the SLAP protein, suggesting that SLAP functions as a dynamic regulator of FcεRI signaling and allergic responses.

## Results

### SLAP Negatively Regulates FcεRI-Dependent Degranulation

**Figure 1:**
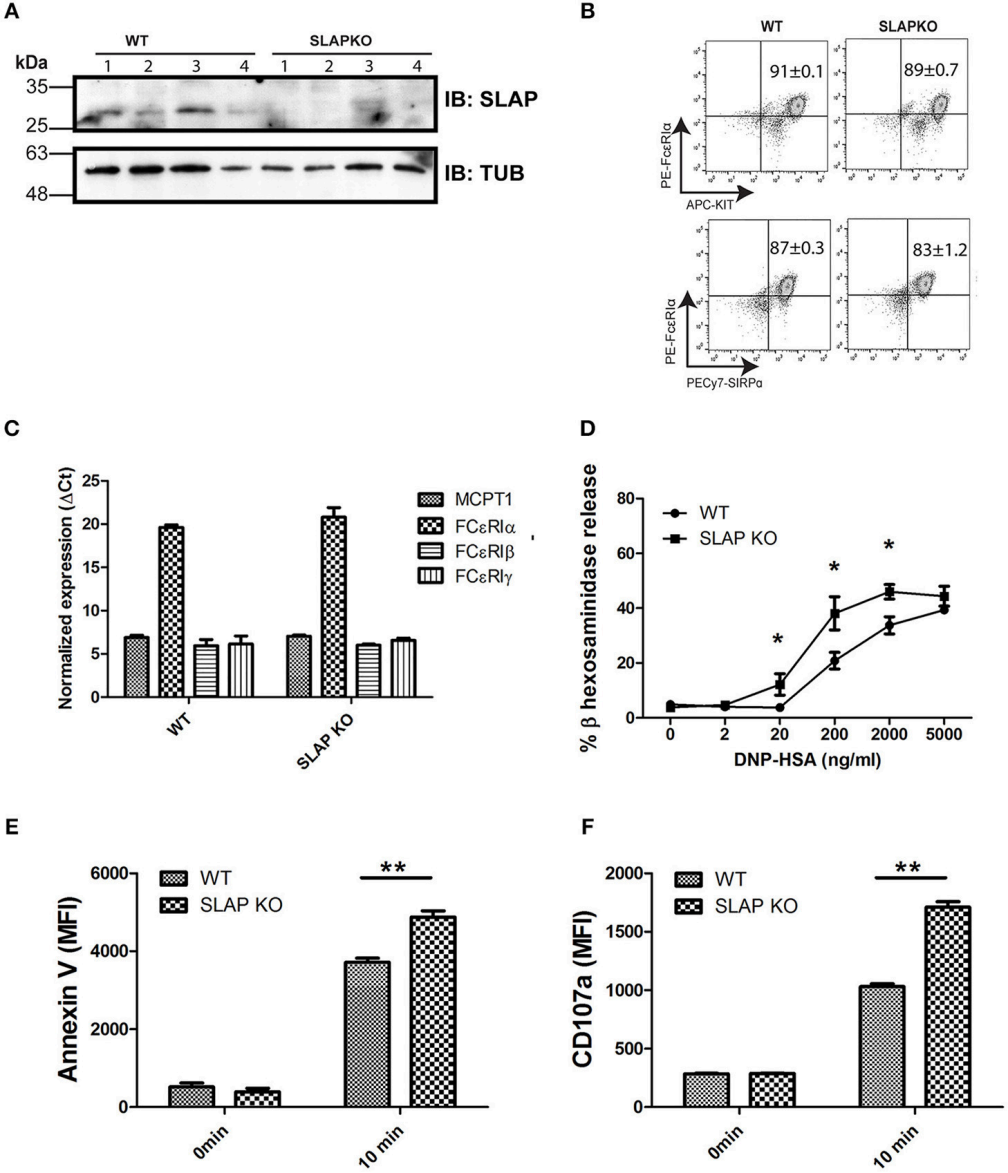
Mature SLAP KO BMMCs show enhanced degranulation upon antigen stimulation. **(A)** Lysates were prepared from WT and SLAP KO BMMCs derived from four different mice and subjected to immunoblotting (IB) with SLAP and Tubulin (Tub) antibodies. Positions of relative mass markers (in kilodaltons) are shown on the left. **(B)** WT and SLAP KO BMMCs were stained with antibodies against cell surface receptors FcεRIα, KIT, and SIRPα. Numbers in dot plots show the mean ± SD values of % double positive (FcεRIα^+^, KIT^+^) or (FcεRIα^+^, SIRPα^+^) of the total cells. Results are from a representative experiment done in triplicate out of three independent experiments (*n* = 3). **(C)** mRNA expression of mast cell markers MCPT1, FcεRIα, FcεRIβ, and FcεRIγ determined by qPCR in mature WT and SLAP KO BMMCs and GAPDH was used as a control to calculate ΔCt values. Data shown are the means ± SD values from a representative experiment done in triplicate, out of two independent experiments (*n* = 2). **(D)** WT and SLAP KO BMMCs were starved and sensitized with anti-DNP IgE for 18 h and stimulated with DNP-HSA at indicated concentrations (0–5,000 ng/ml) for 60 min at 37°C. Line graph depicts β-hexosaminidase released in supernatant post-stimulation and shows as the percentage of the total amount of β-hexosaminidase present in the cells. Data shown are the means ± SD values from a representative experiment done in triplicate, out of three independent experiments (*n* = 3) which showed similar results. A significant difference was observed between genotypes (Student’s *t-*test; *P < 0.05). **(E,F)** WT and SLAP KO BMMCs were sensitized with anti-DNP IgE and stimulated with DNP-HSA (20 ng/ml) for 10 min at 37°C. Bar graph showing the median florescence intensity (MFI) of **(E)** Annexin V and **(F)** CD107a staining of sensitized WT and SLAP KO BMMCs at 0 min and after 10 min of stimulation. Data shown are the means ± SD values from a representative experiment done in triplicate, out of three independent experiments (*n* = 3). (Student’s *t*-test; **P < 0.01).

To investigate the role of SLAP in the regulation of mast cell (MC) functions, bone marrow-derived mast cells (BMMCs) from Balb/C (WT) and SLAP knockout (KO) mice were generated. Immunoblot analysis of mature BMMCs from SLAP KO shows a complete absence of the SLAP protein compared to WT BMMCs (Figure 1A). Since the role of SLAP is well-characterized in the regulation of surface receptor expressions in other immune cell types (13, 18), we investigated the surface expression of characteristic mast cell receptors. FcεRI, cKit (CD117) and SIRPα (CD172a) showed comparable expression levels in both genotypes (*n* = 3) (Figure 1B). Mast cell protease-1 (MCPT-1), FcεRIα, FcεRIβ, and FcεRIγ showed similar mRNA expression levels in MC from WT and SLAP-KO, as determined by quantitative PCR (Figure 1C). This suggested that SLAP KO BMMCs do not have any maturation defect. To understand the role of SLAP in the IgE (FcεRI) receptor-mediated MC function, we measured the FcεRI-mediated degranulation response post-stimulation with the antigen, DNP-HSA. SLAP KO BMMCs showed an enhanced FcεRI mediated degranulation response in a dose dependent manner, compared to control BMMCs based on β-hexosaminidase release (Figure 1D). To confirm the enhanced degranulation of SLAP KO MCs, cell surface phosphatidylserine externalization was measured using annexin V staining on degranulated mast cells as previously described (19). SLAP KO BMMCs showed a significantly higher level of annexin V staining upon IgE cross-linking, as compared to WT cells (Figure 1E). In line with this finding, surface expression of CD107a (LAMP-1), a marker of granular exocytosis (20), was also significantly higher in the SLAP KO BMMCs post-stimulation (Figure 1F). Collectively, these results provide evidence that SLAP is a negative regulator of FcεRI-dependent MC degranulation.

### Enhanced FcεRI-Mediated Cytokine Release in SLAP KO BMMCs

**Figure 2:**
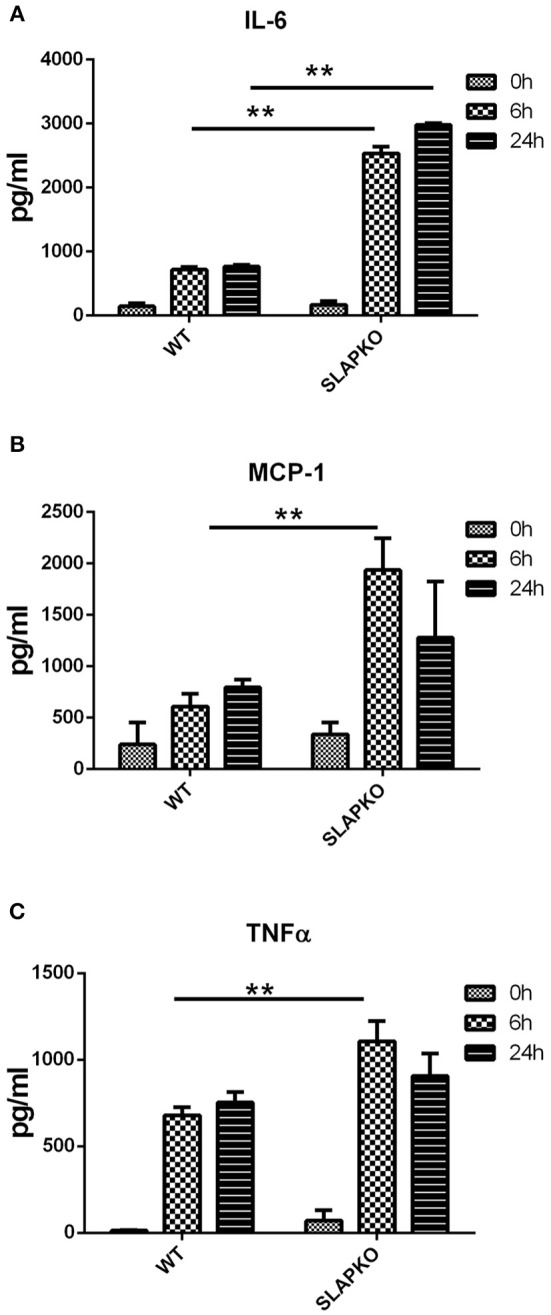
SLAP KO BMMCs shows elevated levels of IgE induced cytokine release. BMMCs from WT and SLAP KO mice were sensitized with anti-DNP IgE and stimulated with DNP-HSA (10 ng/ml) for 0, 6, and 24 h at 37°C. Bar graph showing amount (pg/ml) of secreted **(A)** IL-6; **(B)** MCP-1; and **(C)** TNFα in collected cell supernatants at indicated times as determined by ELISA. Data in **(A–C)** show the means ± SD of a representative experiments done in triplicate, out of three independent experiments (*n* = 3). (Student’s *t-*test; **P < 0.01).

In addition to a rapid degranulation response, FcεRI mediated signaling also regulates the late phase reaction of cytokine secretion via the *de novo* synthesis of cytokines (21). To investigate the role of SLAP in cytokine secretion, BMMCs were sensitized with anti-DNP IgE and stimulated with DNP-HSA (10 ng/ml) for 6 and 24 h, supernatants were collected, and cytokine levels were determined by ELISA. The levels of secreted IL-6, MCP-1, and TNFα were significantly higher in SLAP KO BMMCs at 6 h compared to the WT control (Figures 2A–C) indicating that SLAP negatively regulates cytokine production post-DNP-HSA stimulation.

### SLAP Regulates IgE-Dependent Late Phase Passive Cutaneous Anaphylaxis Response

**Figure 3:**
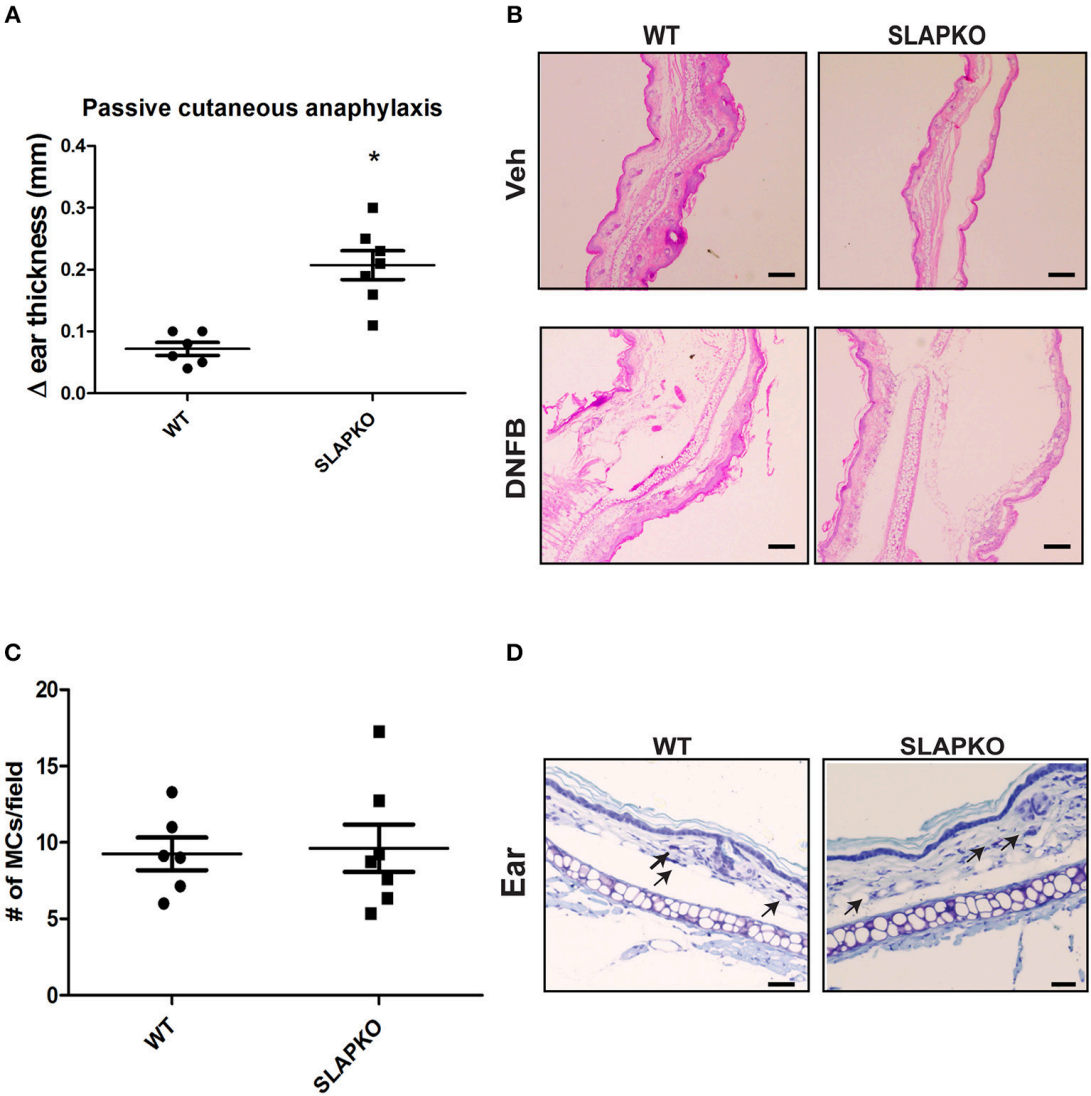
SLAP KO mice show enhanced passive cutaneous anaphylaxis reaction. **(A)** WT (6 mice) and SLAP KO mice (7 mice) were sensitized by tail vein injection with 2 μg anti-DNP–IgE 48 h prior to topical application of DNFB (0.2% [wt/vol]) to the left ear or vehicle (acetone-olive oil, 4:1) to the right ear. After 24 h, mice were culled, and changes in the thicknesses (Δ ear thickness) of DNFB-treated and vehicle-treated ears were recorded with digital calipers. Dot plot showing pooled data of Δ ear thickness from six WT and seven SLAP KO mice, where each dot represents a data from a single mouse with means as a horizontal line and SD values as vertical line. A significant difference was observed between genotypes (Student’s *t*-test; *P < 0.05). **(B)** Representative image of histological section of ear skin from WT and SLAP KO mice treated with either vehicle or DNFB and stained with H&E. Scale bar, 200 μm. **(C)** Dot plot representing the pooled data of the average number of mast cells per field (10–15 fields/ear; from six WT and seven SLAP KO mice where each dot represents data from a single mouse). The horizontal lines in the dot plots represent the mean, and vertical lines are SD values calculated from pooled data. No significant difference was observed between the genotypes. **(D)** Histological section of ear skin from WT and SLAP KO mice were stained with toluidine blue. Mast cells in tissue sections are shown by arrows. Scale bar, 200 μm.

To further characterize the role of SLAP in IgE-mediated mast cell function, we conducted a passive cutaneous-anaphylaxis assay in ear skin as previously described (22–24). WT and SLAP KO mice were sensitized with the IgE antibody for 48 h followed by the topical application of dinitrofluorobenzene (DNFB) (left ear) or vehicle control (right ear). After 24 h, swelling in DNFB treated ears was observed while no reaction occurred in the vehicle treated ear. SLAP KO mice (*n* = 7) had significantly increased ear skin thickness compared to WT mice (*n* = 6) (Figure 3A). Histological examination of ear tissue sections further confirmed that DNFB application resulted in inflammatory swelling in both genotypes, however, inflammation was more pronounced in the SLAP KO ear skin (Figure 3B). To confirm that the enhanced response of SLAP KO mice was due to mast cell activation, rather than elevated mast cell numbers, histological sections of ear skin from WT and SLAP KO mice were examined. Average mast cell numbers/field were comparable in SLAP KO and WT mice in DNFB treated ear skin (Figures 3C,D). This suggests that SLAP negatively regulates the IgE-mediated cutaneous anaphylaxis functional response but does not impact the viability, expansion or the recruitment of mast cells.

**Figure 4:**
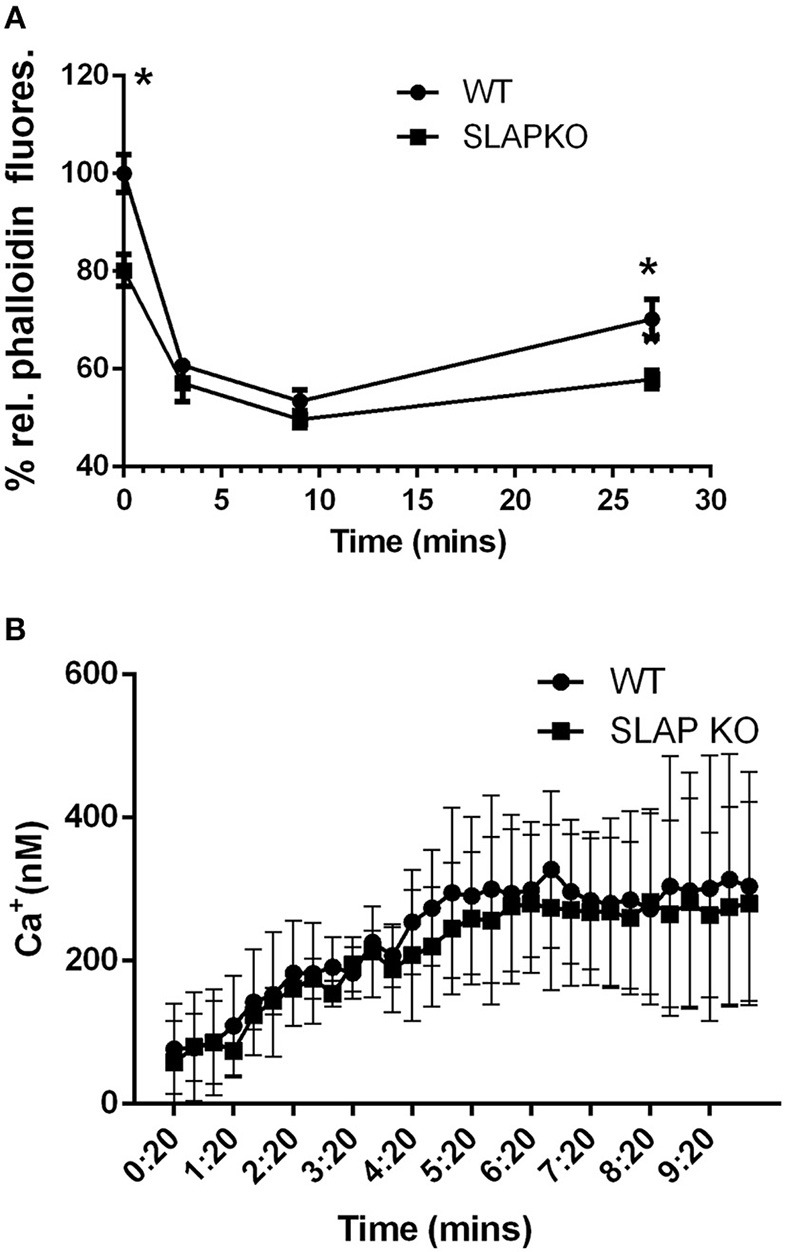
F-actin levels and calcium mobilization in SLAP KO BMMCs. **(A)** BMMCs from WT and SLAP KO genotype were sensitized with Anti-DNP IgE and stimulated with DNP-HSA (20 ng/ml) for indicated times at 37°C. Line graph showing the relative phalloidin fluorescence at indicated times post-stimulation with DNP-HSA (20 ng/ml) in WT and SLAPKO BMMCs where the median fluorescence intensities (MFI) values at all timepoints were shown as a % of MFI values of WT at 0 min timepoint. Data shown are the means ± SD values of one representative experiment done in triplicate, out of total three independent experiments (*n* = 3). A significant difference was observed between genotypes (Student’s *t*-test; *P < 0.05). **(B)** WT and SLAP KO BMMCs were sensitized with Anti-DNP IgE and loaded with Indo-1AM dye and stimulated with either vehicle or DNP-HSA (30 ng/ml). Line graph indicates the amount of intracellular calcium release (nM) at indicated time point. Data represents the means ± SD values of pooled data from three independent experiments (*n* = 3) each of which gave similar results. No significant difference was observed between the genotypes.

To further investigate the impact of SLAP deficiency on MC responses, F-actin polymerization, a key process in mast cell degranulation (25) was examined. Sensitized BMMCs were stimulated with DNP-HSA for the indicated time-points prior to fixation and permeabilization and F-actin content was quantified by phalloidin staining as described previously (26, 27). Flow cytometric analysis showed significantly reduced F-actin levels in SLAP KO BMMCs prior to stimulation (time 0) compared to WT BMMCs (Figure 4A). Following DNP-HSA stimulation, both WT and SLAP KO BMMCs responded similarly, although F-actin remained significantly lower at late time points (27 min). In addition to F-actin polymerization, calcium mobilization in response to FcεRI stimulation contributes to degranulation. Therefore, we loaded the BMMCs with an Indo-1AM ratiometric Ca^2+^ fluorescent indicator and stimulated cells with DNP-HSA or ionomycin in the presence or absence of EDTA. Quantification of the intracellular calcium concentration (nM) showed comparable levels between the genotypes at indicated time points (Figure 4B).

### SLAP Regulates Activation of FcεRI Signaling

**Figure 5:**
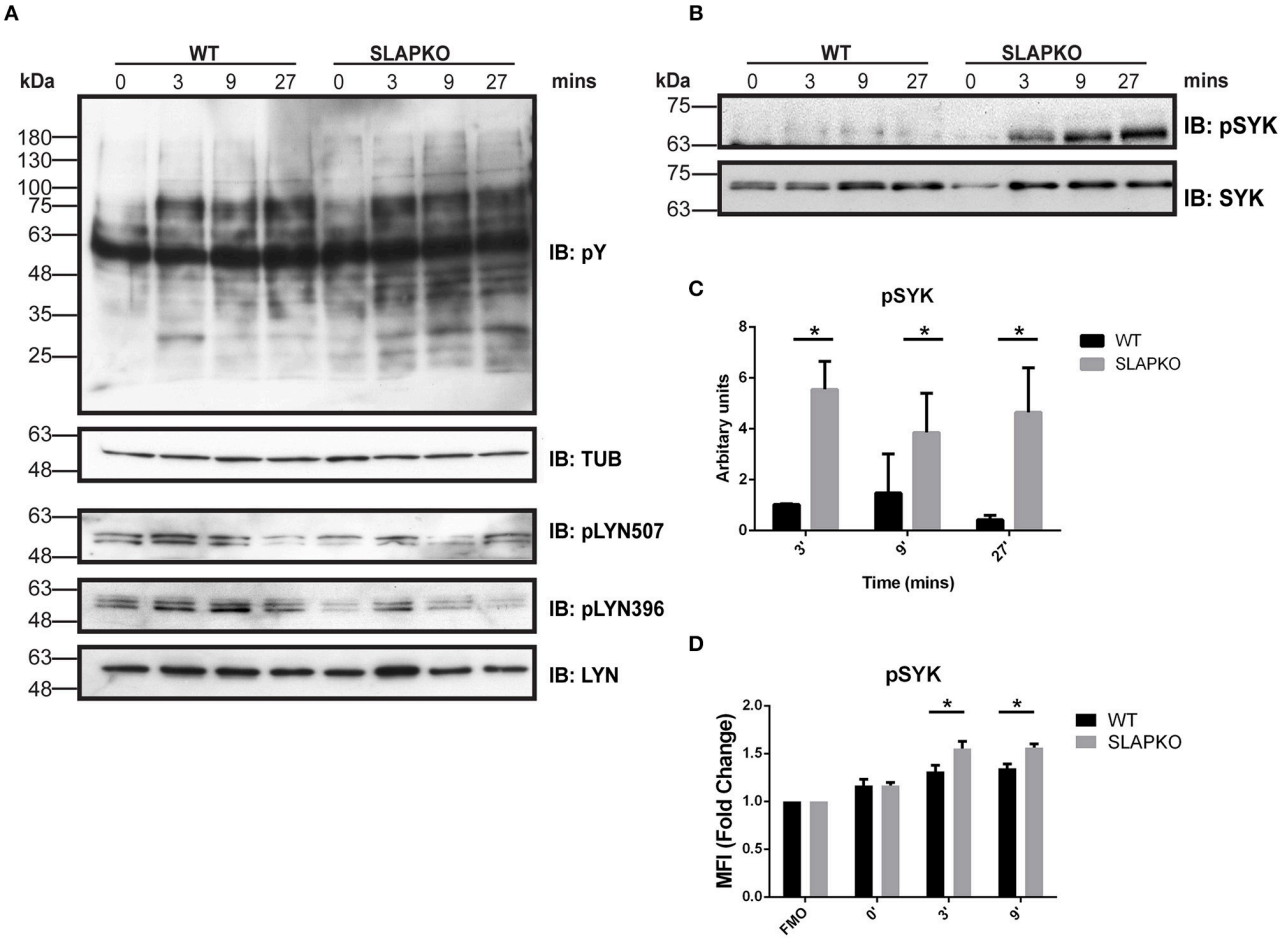
Enhanced Syk signaling in SLAP KO BMMCs. WT and SLAP KO BMMCs were starved and sensitized with Anti-DNP IgE for 18 h and then stimulated with DNP-HSA (50 ng/ml) for indicated time-points. **(A)** Lysates were prepared and subjected to immunoblot (IB) with anti-phosphotyrosine (pY), anti-tubulin (TUB), anti-pY396 Lyn, anti-pY507 Lyn, and anti-Lyn. **(B)** p-Syk Ab, and anti-Syk Abs. Representative immunoblots are shown from three separate experiments (*n* = 3). Positions of relative mass markers in kilodaltons (kDa) are shown on the left. **(C)** Bar graph represents the quantitation of relative pSyk at indicated time points by densitometry quantification. Data (mean ± SD) in bar graph represents the pooled data from three independent experiments (*n* = 3) and compared with Student’s *t*-test; *P < 0.05 to calculate statistical significance. **(D)** WT and SLAP KO BMMCs were starved and sensitized with anti-DNP IgE and stimulated with DNP-HSA for indicated time-points, stained with pSyk Ab and median fluorescence intensity was quantified by phosphoflow cytometry. Bar graphs showing the average of normalized MFI (fold change) of pSYK at indicated time-points compared to respective fluorescence minus one (FMO) control from each genotype. Data (mean ± SD) in bar graph represents the pooled data from three independent experiments (*n* = 3; Student’s *t*-test; *P < 0.05).

To determine the role of SLAP in pathways that regulate degranulation and cytokine responses, we studied the proximal signaling events downstream of FcεRI. IgE-sensitized BMMCs from SLAP KO or WT mice were stimulated with DNP-HSA and phosphorylation of Lyn and Syk was determined by immunoblotting with anti-phosphotyrosine and phospho-site specific antibodies. While there was no change in phosphorylation of Lyn (Figure 5A), enhanced phosphorylation of Syk activation loop tyrosines (Y525/526) was observed in SLAP KO BMMCs at 3, 9, and 27 min after DNP-HSA stimulation compared to WT (Figures 5B,C). Increased phosphorylation of Syk (tyrosine 238) in SLAP KO BMMC was also confirmed using phospho-flow cytometry (Figure 5D). Notably, SLAP KO cells showed a modest increase in the total phosphotyrosine containing proteins, compared to WT BMMCs (Figure 5A).

**Figure 6:**
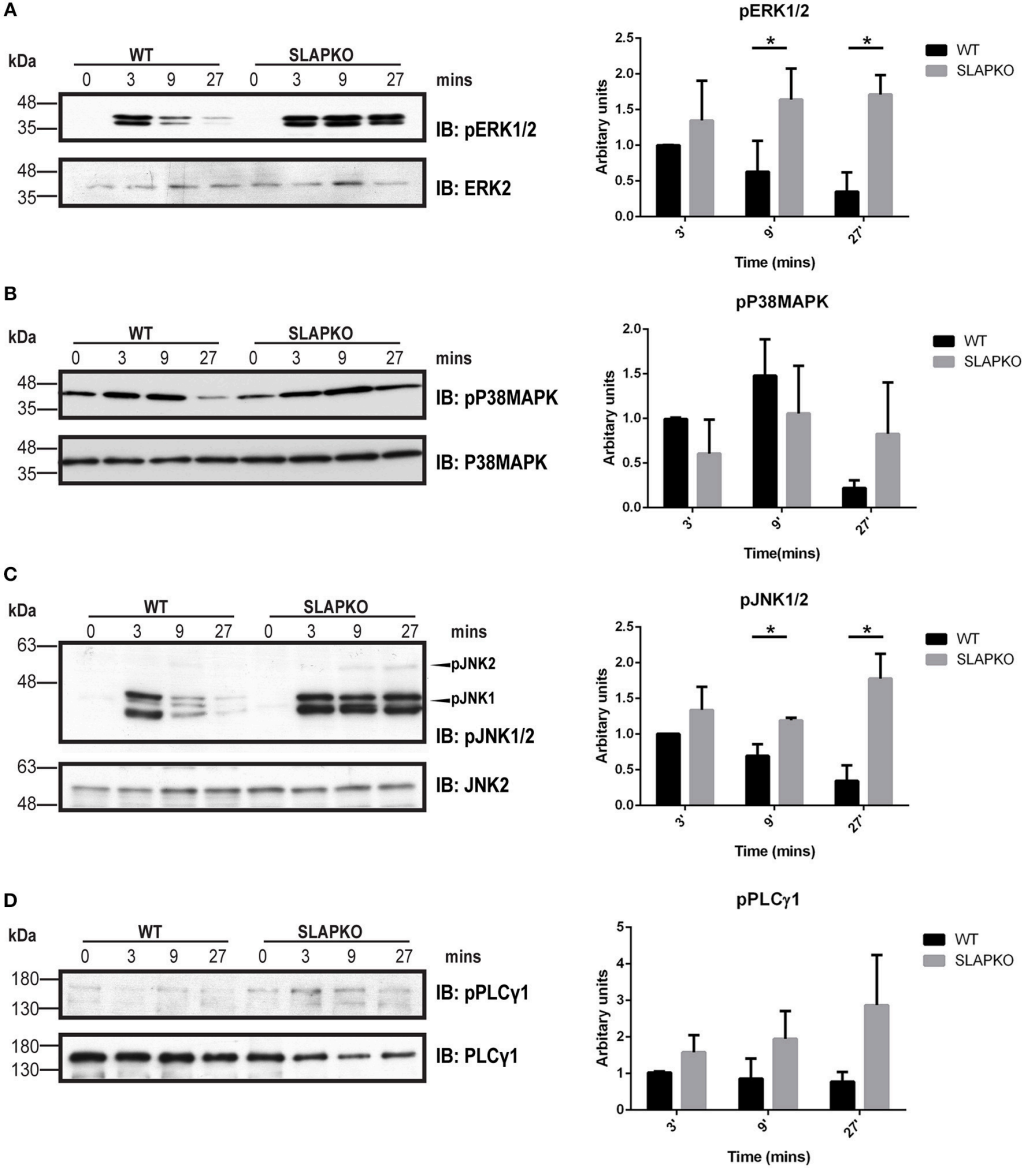
SLAP perturbs the MAPK signaling downstream of FcεRI. WT and SLAP KO BMMCs were starved and sensitized with anti-DNP IgE for 18 h and then stimulated with DNP-HSA (50 ng/ml) for indicated time-points. **(A)** Lysates were prepared and subjected to immunoblot (IB) with anti-phospho-ERK1/2 (pERK1/2), and ERK2 Abs, **(B)** anti-p-P38MAPK, and P38MAPK Abs **(C)** anti-pJNK1/2 and JNK2 **(D)** anti-pPLCγ1, and PLCγ1 Abs. Representative immunoblots of three separate experiments are shown (*n* = 3). Positions of relative mass markers in kilodaltons (kDa) are shown on the left. Bar graph represents the quantitation of relative levels of indicated phospho-proteins at given time points by densitometry quantification. Data (mean ± SD) in bar graph represents the pooled data from three independent experiments (*n* = 3) and compared with Student’s *t*-test; *P < 0.05.

**Figure 7:**
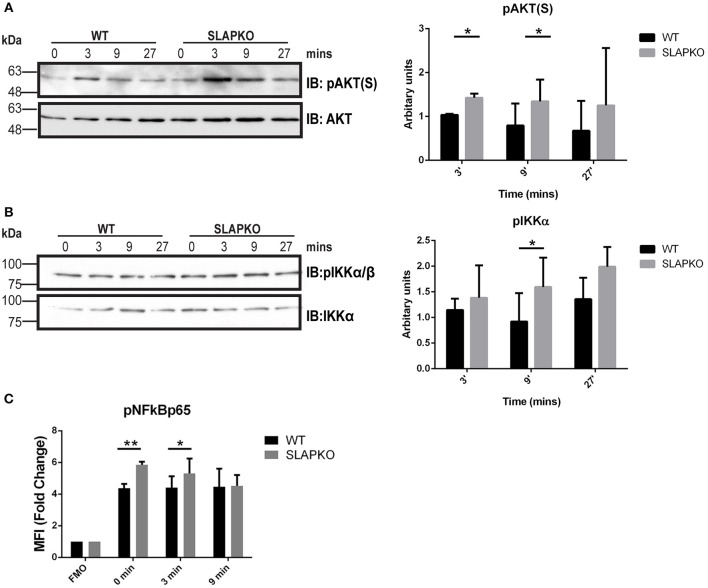
SLAP negatively regulates AKT- NFκB signaling downstream of FcεRI. WT and SLAP KO BMMCs were starved and sensitized with anti-DNP IgE for 18 h and then stimulated with DNP-HSA (50 ng/ml) for indicated time-points. **(A)** Lysates were prepared and subjected to immunoblot (IB) with anti-phospho-AKT serine 473 [pAKT(S)], and AKT Abs, **(B)** anti-p-IKKα/β, and IKKα Abs. Representative immunoblots are shown from three separate experiments (*n* = 3). Positions of relative mass markers in kilodaltons (kDa) are shown on the left. Bar graph represents the quantitation of relative levels of indicated phospho-proteins at given time points by densitometry quantification. Data (mean ± SD) in bar graph represents the pooled data from three independent experiments (*n* = 3) and compared with Student’s *t*-test; *P < 0.05. **(C)** WT and SLAP KO BMMCs were starved and sensitized with anti-DNP IgE and stimulated with DNP-HSA for indicated time-points, stained with pNFκBp65 Ab and median fluorescence intensity was quantified by phosphoflow. Bar graphs showing the average of normalized MFI (fold change) in phosphorylation of NFκB65 at indicated time-points compared to respective fluorescence minus one (FMO) control from each genotype. Data (mean ± SD) in bar graph represents the pooled data from three independent experiments (*n* = 3; Student’s *t*-test; *P < 0.05; **P < 0.01).

Activation of Syk leads to phosphorylation of the membrane adaptor protein LAT, and subsequent recruitment and activation of multiple downstream pathways including Ras-ERK-MAPK and AKT-IKK-NFκB pathways to regulate cytokine and chemokine production (28). Enhanced phosphorylation of ERK1/2 was observed in SLAP deficient BMMCs, and this was most pronounced at later time points, evidence of sustained activation of downstream signaling from SYK and LAT (Figure 6A). While there was no significant difference in p38MAPK phosphorylation (Figure 6B), sustained activation of JNK1/2 was evident in SLAP deficient MCs (Figure 6C). In keeping with the observation that SLAP KO BMMC do not show alterations in calcium mobilization, no significant change in PLCγ1 phosphorylation post-stimulation was observed in SLAP KO BMMCs (Figure 6D). SLAP KO BMMCs also had enhanced AKT phosphorylation, particularly at early time points after antigen stimulation (Figure 7A). Similarly, a modest increase in phosphorylation of IKK and the downstream transcription factor NFkB was apparent at early time points (Figures 7B,C). Collectively, these results indicate that SLAP negatively regulates antigen induced FcεRI signaling to Syk, and its downstream effectors, ERK, JNK, and AKT.

### Impaired Ubiquitination and Internalization of FcεRI in SLAP Deficient MCs

**Figure 8:**
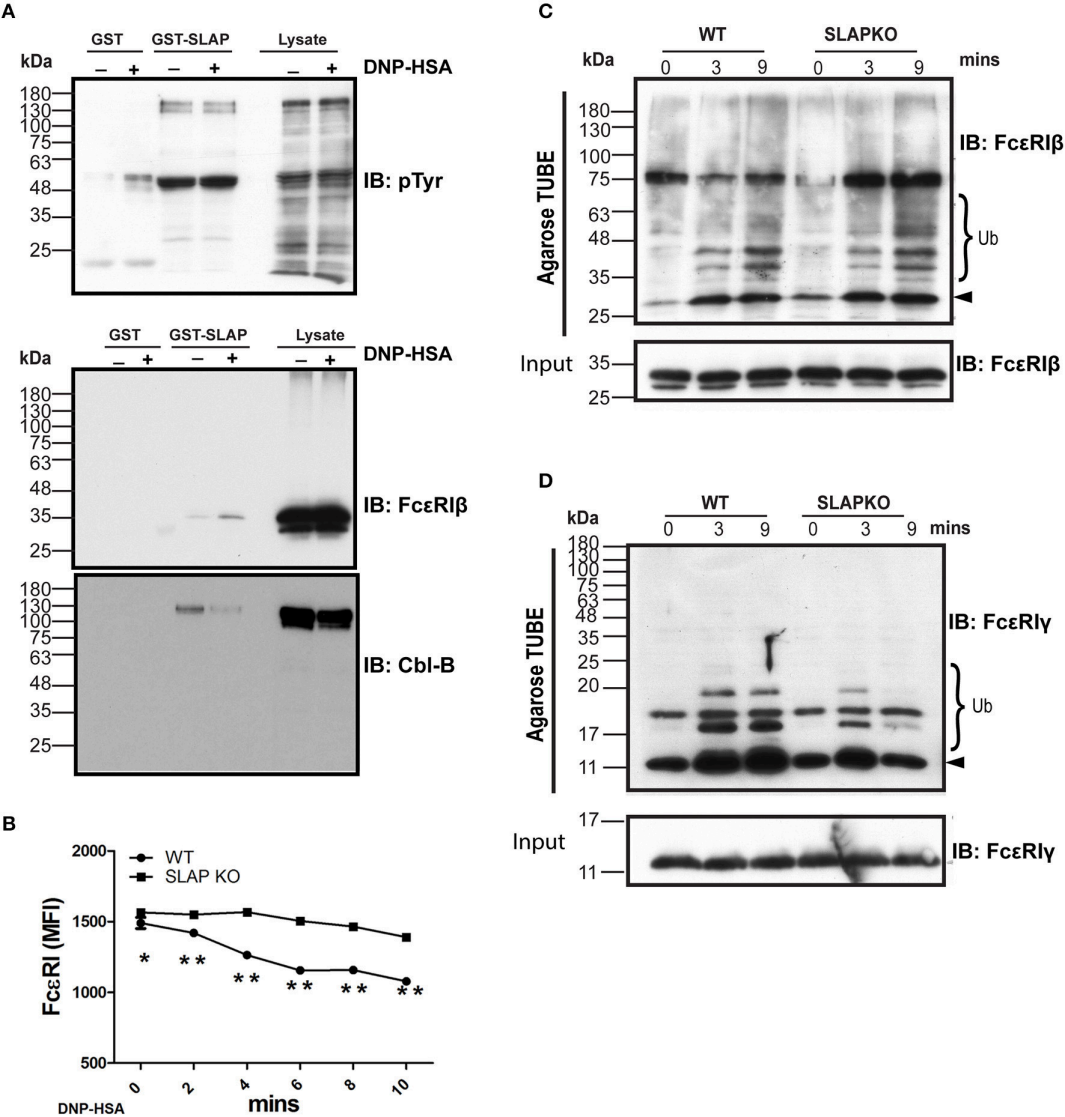
SLAP interacts with FcεRIβ chain and Cbl-B and regulates FcεRI internalization and ubiquitination. **(A)** GST-SLAP fusion protein was incubated with RBL-2H3 lysates and bound proteins were analyzed by immunoblot with anti-phosphotyrosine (pY), anti-FcεRIβ chain, and Cbl-B Abs. Results are representative of three separate experiments (*n* = 3). Positions of relative mass markers in kilodaltons (kDa) are shown on the left. **(B)** Sensitized WT and SLAP KO BMMCs were stimulated with DNP-HSA (20 ng/ml) for the indicated time-points and stained with anti-IgE antibody to quantify surface level of FcεRI receptor. Line graph shows the MFI of FcεRI at indicated time points. Data shown are the means ± SD values from a representative experiment done in triplicate, out of three independent experiments (*n* = 3). A significant difference was observed between genotypes (Student’s *t*-test; *P < 0.05; **P < 0.01). Lysates obtained from sensitized and stimulated WT and SLAP KO BMMCs for indicated times were incubated with ubiquitin binding domain linked agarose (Agarose TUBE) to pull down ubiquitylated proteins. Representative immunoblots showing the ubiquitinated **(C)** FcεRIβ, and **(D)** FcεRIγ chains at indicated times. Results are representative of two independent experiments (*n* = 2). Positions of relative mass markers in kilodaltons (kDa) are shown on the left. Ub indicates the ubiquitinated species and arrowhead shows the indicated protein band at the respective molecular weight.

Previously, SLAP has been shown to regulate the surface expression and down regulation of receptors such as TCR and BCR via recruitment of the ubiquitin ligase Cbl (13, 29, 30). Therefore, one possible mechanism to explain the enhanced signaling in SLAP deficient BMMCs is due to impaired Cbl function in the absence of SLAP. Prior studies have indicated that Cbl-b is the dominant Cbl family member expressed in BMMC where it has been demonstrated to regulate the surface expression and signaling downstream of FcεRI (10). To assess whether SLAP loss might have effects on Cbl-b function or its activity, we first tested whether SLAP interacts with Cbl-b. GST-SLAP fusion proteins were incubated with RBL-2H3 cell lysates and bound proteins analyzed by immunoblot. GST- SLAP bound to several tyrosine phosphorylated proteins including Cbl-b and FcεRIβ in both un-stimulated and DNP-HSA stimulated cells (Figure 8A). To investigate whether SLAP affects the surface expression of FcεRI upon stimulation with antigen, IgE-sensitized BMMCs were stimulated with DNP-HSA (20 ng/ml) and cell surface levels of FcεRI were determined by flow cytometry. Quantification of MFI as a measure of receptor surface expression levels revealed that SLAP KO BMMCs retained significantly higher levels of the high affinity IgE receptor compared to WT at all time-points post-stimulation (Figure 8B), suggesting impaired internalization of FcεRI. The association of SLAP and Cbl-b and altered FcεRI internalization in SLAP deficient cells could suggest that either the adaptor or ubiquitin ligase function of Cbl-b is defective in the absence of SLAP. In the RBL-2H3 cell line, both FcεRIβ and FcεRIγ are ubiquitinated following receptor aggregation and Cbl overexpression has been shown to promote ubiquitination of both subunits and enhances receptor internalization (31, 32). To test the impact of SLAP deficiency on FcεRI ubiquitination, stimulated cell lysates from wild type or SLAP KO BMMCs were incubated with ubiquitin binding domain linked agarose (agarose-TUBE) and bound proteins were detected by immunoblot. Ubiquitinated species of both FcεRIβ and FcεRIγ were observed at 3- and 9-min following stimulation. While ubiquitination of FcεRIβ appeared unaffected in the absence of SLAP (Figure 8C), recovery of ubiquitinated species of FcεRIγ was reduced in SLAP deficient compared to wild type cells (Figure 8D). Altogether these data support a model in which SLAP is important for recruitment of Cbl-b to FcεRI, ubiquitination of FcεRIγ and receptor down regulation.

### Activation of Human Basophils Regulates Expression of SLAP

**Figure 9:**
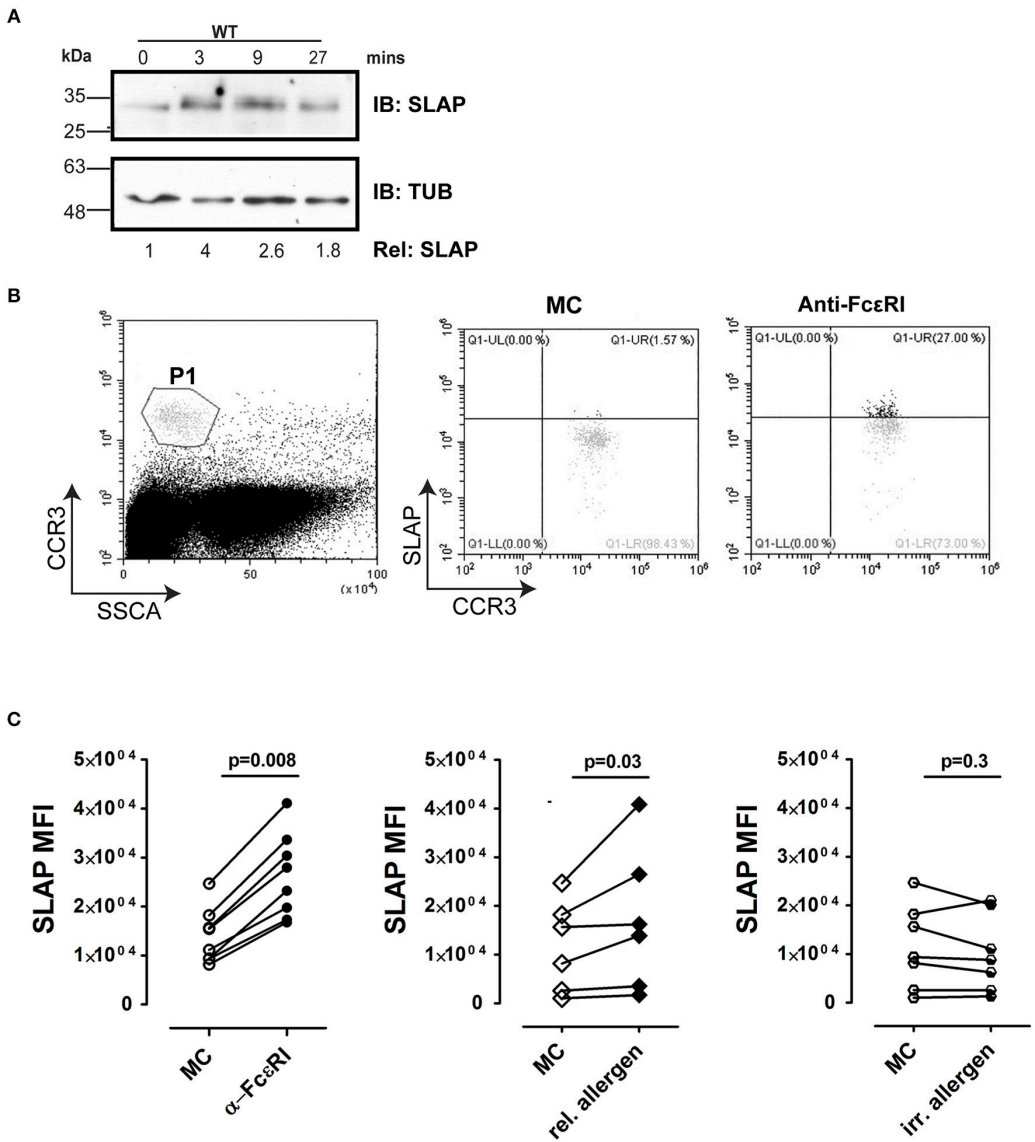
SLAP expression is regulated by FcεRI signaling in BMMCs and human basophils. **(A)** WT and SLAP KO BMMCs were starved and sensitized with anti-DNP IgE for 18 h and then stimulated with DNP-HSA (50 ng/ml) for indicated time-points. Lysates were prepared and subjected to immunoblot (IB) with anti-SLAP, and anti-tubulin (TUB) Abs. Results are representative of three separate experiments (*n* = 3). Densitometry was performed to determine the relative SLAP protein levels (the WT level at 0 min was set as 1.0). Positions of relative mass markers in kilodaltons (kDa) are shown on the left. **(B)** Gating strategy for basophils in human samples. Basophil were identified as CCR3^hi^/SSC^low^, indicated as P1 in dot plot. SLAP expression was quantified in P1 population by intracellular staining with anti-SLAP and flow cytometry. **(C)** Basophils from atopic donors were incubated with human Fc blocking antibody and then treated with medium control (MC) or stimulated with anti-FcεRI antibody [positive control, (*n* = 8), relevant (allergic, *n* = 4)] or irrelevant allergen (*n* = 5) and stained with anti-SLAP Ab. The median fluorescence intensity of intracellular staining of SLAP with and without stimulation of each donor sample is shown. Wilcoxon matched-pairs signed-rank test was used to calculate statistical significance.

A previous study in the RBL-2H3 cell line demonstrated that SLAP expression is upregulated in response to antigen stimulation (33). In agreement, the levels of SLAP protein detected by immunoblotting increased following the antigen stimulation of murine BMMCs (Figure 9A). To further assess the contribution of SLAP in IgE receptor signaling, we determined intracellular SLAP protein expression in peripheral blood derived human basophils, upon anti-FcεRI or allergen stimulation. Basophils derived from eight human donors, uniformly up-regulated SLAP expression upon FcεRI receptor cross-linking (Figures 9B,C). Furthermore, the same response was demonstrated at an allergen specific level. Basophils from food allergy patients stimulated with relevant food allergen showed a significant increase in SLAP protein levels similar to that observed upon FcεRI cross-linking, whereas stimulation with a clinically irrelevant food allergen resulted in no change in SLAP levels (Figure 9C). All experiments were controlled for the degranulation of basophils (CD63 expression) in response to FcεRI cross linking antibody and the allergen. These results indicate that FcεRI stimulation modulates SLAP expression in human basophils and suggests that SLAP has a role in modulating allergic responses.

## Discussion

MCs are key drivers in mounting and propagating allergic diseases such as allergic rhinitis, conjunctivitis, atopic dermatitis, food allergy, allergic asthma, and IgE-mediated anaphylaxis (34). Sensitizing mast cells by binding IgE to FcεRI on MCs is considered the crucial event conditional for an allergic response. Binding of IgE to the FcεRI has been shown to further increase the expression of FcεRI in human and mouse-derived MCs (35, 36). Consistently, atopic dermatitis patients with an elevated amount of circulating IgE showed enhanced FcεRI on the surface of MCs and basophils (37, 38). Therefore, a balanced expression of FcεRI and controlled downstream signaling are determinants of a protective immune response vs. detrimental allergic diseases. Thus, understanding of the critical positive and negative regulators of IgE- FcεRI signaling is required to target this balance for therapeutic purposes.

Here, we report that SLAP is a negative regulator of FcεRI signaling and late phase cytokine response in primary bone marrow derived mast cells (BMMC). SLAP null BMMCs showed increased degranulation and cytokine production in response to antigen stimulation and SLAP KO mice have an enhanced subcutaneous anaphylaxis response compared to the WT control. Consistently, we also observed effects of SLAP loss on activation of Syk, ERK and JNK, as well as NFκB signaling downstream of FcεRI. A previous study reported that SLAP knock down in the rat basophilic leukemia cell line RBL-2H3, had similar effects on signaling downstream of FcεRI, but had no effect on degranulation (33). However, the RBL-2H3 carries a constitutive active mutation in the kinase domain of c-Kit (39) and therefore activated c-Kit signaling may have masked the effects of partial knockdown of SLAP expression on degranulation.

The increased cytokine production observed in SLAP deficient MCs is explained by enhanced AKT and MAPK signaling activating NFκB and additional transcription factors including Elk1 and NFAT leading to cytokine response (28, 40, 41). Enhanced degranulation and cytokine secretion likely explain the *in vivo* phenotype of SLAP KO mice in response to local MC-mediated anaphylaxis challenges.

Our investigation revealed reduced levels of F-actin in SLAP KO MCs compared to WT but a comparable reduction in response to FcεRI crosslinking. Mast cell granule exocytosis and subsequent degranulation is dependent upon actin cytoskeletal rearrangement triggered by antigen stimulation (42). In addition, treatment of BMMCs with a drug that perturbs F-actin polymerization showed enhanced degranulation suggesting the reciprocal relation of F-actin level and degranulation (20). Therefore, the reduced F-actin levels in SLAP KO MCs may lower the stimulation threshold required to release granules and partially contribute to the enhanced degranulation phenotype. The mechanisms that underlie reduced F-actin levels in SLAP KO BMMCs are unclear. A potential explanation is that the function of Cbl-b in regulation of F-actin polymerization via the actin modulatory protein, Wiskott-Aldrich syndrome protein (WASP) observed in T cells, might also be defective in SLAPKO BMMCs (43). Further investigation to address WASP degradation kinetics in the IgE receptor pathway are required to address the potential role of SLAP in actin cytoskeletal dynamics in MCs.

Reduced FcεRI internalization and enhanced signaling in SLAP deficient MCs were two important findings of this study. Previously, Cbl-b null MCs were shown to have reduced endocytosis of FcεRI along with enhanced signaling, degranulation, and cytokine production, but no defects in calcium mobilization post-antigen stimulation were observed (10, 44). Importantly, Cbl-b functions in this context are both RING finger domain dependent and independent. For example, while regulation of FcεRI signaling is largely dependent on Cbl-b ubiquitin ligase function, activation of inflammatory cytokine production is independent of the Cbl-b RING finger domain (44, 45). SLAP KO MCs display a very similar phenotype to the Cbl-b null MCs suggesting that they act through related mechanisms to regulate IgE-FcεRI pathways. SLAP binding to Cbl-b, decreased FcεRI internalization and FcεRIγ ubiquitination in SLAP KO cells post-antigen stimulation, suggest a role of SLAP in the function of Cbl-b. One model to explain these results is that SLAP binding to FcεRIβ plays a role in the recruitment or localization of Cbl-b to the FcεRI receptor. Receptor recruitment would then promote phosphorylation of Cbl-b and increase both its ubiquitination and adaptor functions. This model could account for the changes in FcεRI receptor mediated endocytosis, signaling and degranulation observed in SLAP deficient MC as a consequence of defective adapter and ubiquitin ligase activity of Cbl-b.

Clinical successes, by reducing the amount of circulating IgE with antibodies such as omalizumab, in patients with allergic disorder suggested the importance of regulating IgE- FcεRI pathways (46–49). In addition, small molecule inhibitors that perturb the IgE-FcεRI signaling showed marked improvement in preclinical allergic disease models (50–52). Such therapeutics highlight the importance of regulating the signaling pathways in controlling allergic disease onset and progression. Exploiting the negative regulators of downstream signaling of FcεRI by enhancing their function leading to termination of signaling pathways could also be an alternative strategy. For example, a previous study showed that small molecules leading to enhanced expression of SHP1 in cancer lines and dampened downstream signaling to STAT3 (53, 54) could also be employed in allergic diseases. Our study highlights the role of SLAP as a negative regulator of FcεRI-mediated signaling. This suggests that enhancing the function of SLAP or even Cbl-b could have therapeutic implications.

## Materials and Methods

### Mice

SLAP^−^ Balb/c (SLAP KO) have been described previously (12, 14) and Balb/c (wild type:WT) mice were bred and maintained under institute guidelines at the Toronto Centre for Phenogenomics. Experiments were performed on both male and female mice at 10–12 weeks of age. All studies and experimental procedures were approved by the Toronto Centre for Phenogenomics Animal Care Committee.

### Bone Marrow Derived Mast Cell (BMMC) Culture

Suspended BMMC cultures were derived as previously described (24, 55). Briefly, bone marrow cells from SLAP KO and WT mice were cultured for 4 weeks to generate BMMC cultures in BMMC growth medium [IMDM, 16% (v/v) Fetal calf serum (ATCC: 30-2030), 1% (v/v) antimicrobial-antimycotic solution (Invitrogen), 1 mM sodium pyruvate (Invitrogen), 1% (v/v) minimum non-essential amino acids (Invitrogen), 2% (v/v) conditioned medium from X63-IL-3 cells and 50 μM α-monothioylglycolate (Sigma-Aldrich)]. Maturation of BMMCs were determined by analyzing the expression of surface FcεRIα and Kit receptor using Life Technologies Attune® acoustic focusing cytometer. BMMC cultures expressing >90% double positive (FcεRIα^+^/Kit^+^) were considered mature and further used for experiments. The following flow antibodies were used: Biolegend anti- IL-6 (APC; clone MP5-20F3), cKIT(CD117, APCCy7; clone 2B8), IgE (FITC; clone 23G3), CD45 (PE-Cy7;clone 30-F11), SIRPα (CD172a, PE-Cy7; clone P84), Rat IgG2b κ (Isotype, APC-Cy7; clone RTK4530), Rat IgG1, κ (Isotype, APC; clone RTK2071), Rat IgG1, κ (Isotype, PE-Cy7; clone RTK2071), BD Bioscience anti- TNFα, (PE;clone MP6-XT22), or ebiosciences anti-CD16/CD32 (FcBlock, purified, clone 93).

### IgE Receptor Stimulation of Mast Cells and Cell Lysis

Mature BMMCs (10^7^ cells/time point) were starved overnight in starvation medium [IMDM, 2% (v/v) Fetal calf serum (ATCC: 30-2030), 1% (v/v) antimicrobial-antimycotic solution (Invitrogen), 1 mM sodium pyruvate (Invitrogen), 1% (v/v) minimum non-essential amino acids (Invitrogen), and 50 μM α-monothioylglycolate (Sigma-Aldrich)] with 20% (v/v) anti-DNP-IgE conditioned medium (SPE7 clone; ≈1 μg/ml) for 18 h. Sensitized BMMCs were washed in Tyrode’s buffer [10 mM HEPES (pH 7.4), 130 mM NaCl, 5 mM KCl, 1.4 mM CaCl_2_, 1 mM MgCl_2_, 5.6 mM glucose, 0.1% BSA] twice. Mast cells were stimulated with either vehicle control or 30 ng/ml dinitrophenyl-human serum albumin (DNP-HSA; Santacruz Biotechnology) in Tyrode’s buffer at 37°C for indicated timepoints and reactions were by adding ice-cold PBS. Soluble cell lysates (SCL) were prepared in Phospho-lipase C buffer [PLC buffer; 50 mM HEPES (pH 7.5), 150 mM NaCl, 105 Glycerol, 1 mM EGTA, 1% (v/v) Triton-X, 1.5 mM Mgcl_2_, complete protease inhibitor cocktail (Roche), and phosphatase inhibitor (Cell Signaling Technology)] at 12,000 g for 15 min at 4°C.

### Degranulation Assay

Degranulation assays were performed as described previously (56). Briefly, BMMCs of SLAP KO and WT genotypes (1 × 10^6^ cells/ml) were starved and sensitized with 20% (v/v) anti-DNP-IgE conditioned medium for 18 h, and washed twice with warm Tyrode’s buffer. In 96-well plates, BMMCs (6.25 × 10^4^ cells/well) were stimulated for 1 h in Tyrode’s buffer supplemented with vehicle control, DNP-HSA in increasing concentrations at 37°C. Supernatants and cell lysates prepared in Tyrode’s buffer supplemented with 0.5% Triton X-100 were incubated with p-nitrophenyl N-acetyl-β-D-glucosamide (1.3 mg/ml) for 1 h at 37°C and reactions were stopped with the addition of 0.2 M Glycine buffer (pH 10.7). A colorimetric assay was performed to measure β-hexosaminidase activity to calculate the % release (supernatant) determined as a percentage of the total activity in the supernatant and pellet.

### Immunoblot Analysis

Samples were boiled for 10 min and resolved on 8–12% SDS-PAGE gels. The following antibodies were used: Rabbit anti-Lyn (2796), rabbit anti-phospho-Lyn 507 (2731), rabbit anti-Syk (2712), rabbit anti-phospho-Syk (2710), rabbit anti-Cbl-b (9498), rabbit anti-AKT (2731), rabbit anti-phospho-AKT (9271), rabbit anti-PLCγ1 (5690), rabbit anti-phospho-PLCγ1 (2821), rabbit anti-IKKα (2682), rabbit anti-phospho-IKKα/β (2697), rabbit anti-phospho-Erk1/2 (9101), rabbit anti-phospo-P38 MAPK (9211), rabbit anti-p38MAPK (9212), rabbit anti-phospho-JNK1/2 (9251), rabbit anti-JNK (9252) were all from Cell signaling technology. Mouse anti–FcεRIβ (H 5), mouse anti-pTyr (pY99), goat anti-SLAP (C19), and mouse anti-Cbl-b (G1) were from Santacruz Biotechnology, rabbit anti-FcεRIγ (06-727) were from Millipore. Mouse anti-tubulin (T6034, Sigma-Aldrich), and Mouse Anti-ERK2 (Clone 33/ERK2, BD transduction Lab) were also used. Secondary Abs were HRP-conjugated donkey anti-goat IgG (Jackson immunoresearch lab), HRP-conjugated goat anti-mouse IgG, HRP-conjugated goat anti-rabbit IgG (Cell signaling technology).

### Cytokine Release and Intracellular Cytokine Staining

BMMCs (WT and SLAPKO; 5 × 10^6^ cells/ml per sample) were starved and sensitized with 20% (v/v) anti-DNP-IgE conditioned medium (SPE7 clone; ≈1 μg/ml) for 18 h, and then stimulated (2.5 × 10^6^ cells/ml) with either vehicle control or 10 ng/ml DNP-HSA for 6 h at 37°C. Cells supernatants were analyzed to quantify the amount of IL-6, MCP-1, and TNFα by ELISA kits (ebiosciences) as per manufacture instructions.

### Actin Polymerization Assay

IgE sensitized BMMCs (WT and SLAPKO) were stimulated with DNP-HSA (30 ng/ml) for 0, 2, 4, 6, 8, and 10 min and fixed in ice cold 2% paraformaldehyde/5 mM EGTA/5 mM EDTA and subsequently treated with permeabilization buffer (PBS/0.1% saponin/2% BSA). Polymerized actin was quantified with staining for phalloidin (10 nM; molecular probes) and analyzed by flow cytometry as previously described (56, 57).

### Passive Cutaneous Anaphylaxis Assay

WT (*n* = 6) and SLAP KO mice (*n* = 7) mice from two independent experiments were sensitized with 2 μg anti-dinitrophenyl-IgE (Sigma-Aldrich) by i.v. injection. After 48 h, dinitrofluorobenzene [20 μl volume of 0.2% (w/v) of DNFB (Sigma-Aldrich); in acetone-olive oil] was applied on the right ear while the left ear was treated with the vehicle alone as previously described (58). Difference in ear thickness (Δ ear thickness) between DNFB- and vehicle-treated skin was quantified using digital calipers. H&E stained images of DNFB- and vehicle-treated ear skin were acquired with an EVOS FL color imaging system (Thermo Fisher scientific) with 20 × objective (air).

### Phosphoflow Staining

Starved and sensitized BMMCs [WT and SLAP KO (20 × 10^6^ cells)] were washed twice in PBS and then re-suspended in RPMI (Phenol red free) supplemented with 10 mM HEPES 7.4, 1 mM sodium pyruvate and 1% (v/v) minimum non-essential amino acids for 4 h. Subsequently, cells were stimulated in Tyrode’s buffer with vehicle or DNP-HSA (30 ng/ml) for indicated time-points and fixed with 2% paraformaldehyde for 10 min at 37°C, followed by washing with PB (PBS supplemented with 1% bovine serum albumin) buffer twice before permeabilization with cold (−20°C) BD phosphoflow perm buffer III for 30 min. After permeabilization, cells were stained with antibody solution (1:100 dilution) for 30 min at room temperature before analysis on a cytometer. The following BD Bioscience antibodies for phosphoflow analysis were used: pSyk (pY348, PE; clone 1120-722) and pNFkB p65 (S529, PE; clone K10-895.12.5).

### Affinity Purification of Ubiquitinated Proteins

Starved and sensitized BMMCs (WT and SLAP KO; 60 × 10^6^) were incubated with MG132 (25 mM; Merck) and chloroquine (50 mM; Sigma-Aldrich) for 2 h before DNP–HSA stimulation. BMMCs (20 × 10^6^ cells per sample) were lysed in 500 ml ice-cold ubiquitin lysis buffer [50 mM Tris-Cl, pH 7.5, 5 mM EDTA, 10 mM Na_4_P_2_O_7_, 100 mM NaCl, 1% Triton X-100, protease inhibitor, phosphatase inhibitors and 20 μM deubiquitlyase inhibitor (PR-619; lifesensors]. Agarose-Tandem Ubiquitin Binding Entities (TUBEs) (Lifesensors) (20 μl/sample) were incubated with cell lysates at 4°C for 2 h and were then washed three times with cold tris-buffered saline with Tween-20 (TBS-T). Bound ubiquitinated proteins were eluted and resolved with SDS–PAGE, followed by immunoblot with FcεRIβ, and FcεRIγ chain antibodies.

### Quantitative PCR Reaction

Total RNA was isolated using the RNEasy mini kit (Qiagen) and reverse transcribed with the High-Capacity cDNA Reverse Transcription kit (Applied Biosystems) per the manufacturer’s instructions. For quantitative PCR analysis, 1 μL cDNA was combined with SsoAdvanced Universal SYBR Green Supermix (BioRad) and 0.5 μM forward and reverse primers. Reactions were set up in triplicate and run on an Applied Biosystems StepOnePlus thermal cycler, using cycling conditions described by the manufacturer and an annealing temperature of 60°C. Reactions were normalized to a GAPDH expression to calculate ΔCt values. The following primers were used for the qPCR reaction: FcεRIα (For:AGAGCAAACCTGTGTACTTG, Rev:GTGGACTTTCCATTTCTTCC), FcεRIβ (For: AGGCTACCCATTCTGGGGTG, Rev: GGCTGCCTCTCACCAGATAC), FcεRIγ (For: CTCCTTTTGGTGGAAGAAGC, Rev:TGAGTCGACAGTAGAGCAGG), MCPT1 (For:TTCCCTTGCCTGGTCCCT, Rev:GTTTTCCCCCAGCCAGCT).

### Calcium Mobilization

Starved and sensitized BMMCs (WT and SLAP KO; 2 × 10^6^/sample) loaded with Indo-1AM as described previously (59). In 96 well-black plate with clear bottom, cells were stimulated with vehicle, DNP-HSA (20 ng/ml), and ionomycin (5 μM) in presence or absence of EGTA (10 nM). Cells loaded with Indo-1AM were excited with a UV lamp tuned to 338 nm while capturing emissions at 405 and 480 nm every 20 ms at Molecular Devices SpectraMax Gemini EM. The concentration of intracellular free Ca^2+^ was determined as previously (59).

### GST Pull Down Assay

GST or GST-SLAP fusion proteins were purified as described previously (14). Fusion proteins were quantified by SDS-PAGE followed by Coomassie staining and compared with BSA standards. For GST pull down assay, 500 μg of RBL-2H3 protein lysate was incubated with GST or GST-SLAP bound beads (2 μg/sample) for 2 h at 4°C. Beads were washed three times with PLC buffer and samples were resolved on 10% SDS-PAGE gel and immunoblotted with anti- pY, anti- FcεRIβ chain (Santacruz Biotechnology), and anti-Cbl-b (Cell Signaling Technology) antibodies.

### Analysis of SLAP Expression in Human Basophils

Blood samples were obtained from atopic children aged 4–6 years with informed consent and according to the protocol approved by the Research Ethics Board of the Hospital for Sick Children (REB#1000041089). After blocking with Fc block (BD biosciences 564220) cells were subjected to stimulation with stimulation buffer (Bühlmann B-CCR-STB) or anti-FcεRI mAb (Bühlmann B-CCR-STCON), or with relevant (allergic) or irrelevant (not allergic) allergen (100 ng/mL), and stained for the surface marker CCR3 (Biolegend 310708) simultaneously for 15 min at 37°C. Thereafter the reaction was stopped with fix/lysis buffer 5X (BD biosciences 558049) for 10 min at 37°C and cells were treated with permeabilization buffer III (BD biosciences 558050) overnight at −20°C. Cells were then incubated for 40 min at 4°C with mouse anti-SLAP mAb (sc-135802) and for 20 min with Cy3-conjugated goat anti-mouse secondary antibody. SLAP median fluorescence in basophils (*n* = 500; SSC^low^/CCR3^pos^) was quantified on a CytoFLEX flow cytometer (Beckman Coulter).

### Statistical Analysis

For analyzing the flow cytometry data, flowjo 10.0.8 was employed. BMMC phospho-flow data was further analyzed by Cytobank software to quantify the fold change in phospho-profile. A two-tailed unpaired Student’s *t*-test was used for all statistical analyses except the human basophil analysis where a Wilcoxon match paired *t*-test was applied. Graph Pad prism software was used to produce graphs and for the quantification of statistical significance (^*^P < 0.05 and ^**^P < 0.01).

## Ethics Statement

This study was carried out in accordance with institute guidelines at the Toronto Centre for Phenogenomics and experimental protocols were approved by the Toronto Centre for Phenogenomics Animal Care Committee. Blood samples were obtained from atopic children aged 4–6 years with informed consent and according to the protocol approved by the Research Ethics Board of the Hospital for Sick Children (REB#1000041089).

## Author Contributions

NS, MP, SK, DB, and ZP designed, conducted experiments, and analyzed data. CJM and TE designed experiments and analyzed data. NS, MP, TE, and CJM wrote the manuscript.

### Conflict of Interest Statement

The authors declare that the research was conducted in the absence of any commercial or financial relationships that could be construed as a potential conflict of interest.
